# Interface Engineering of Titanium Nitride Nanotube Composites for Excellent Microwave Absorption at Elevated Temperature

**DOI:** 10.1007/s40820-024-01381-w

**Published:** 2024-04-04

**Authors:** Cuiping Li, Dan Li, Shuai Zhang, Long Ma, Lei Zhang, Jingwei Zhang, Chunhong Gong

**Affiliations:** 1 College of Chemistry and Molecular Sciences, Henan University, Kaifeng, 475004 People’s Republic of China; 2 National and Local Joint Engineering Research Center for Applied Technology of Hybrid Nanomaterials, Henan University, Kaifeng, 475004 People’s Republic of China

**Keywords:** TiN nanotubes, Interface engineering, Polarization loss, Impedance matching, Electromagnetic wave absorption performance

## Abstract

Currently, the microwave absorbers usually suffer dreadful electromagnetic wave absorption (EMWA) performance damping at elevated temperature due to impedance mismatching induced by increased conduction loss. Consequently, the development of high-performance EMWA materials with good impedance matching and strong loss ability in wide temperature spectrum has emerged as a top priority. Herein, due to the high melting point, good electrical conductivity, excellent environmental stability, EM coupling effect, and abundant interfaces of titanium nitride (TiN) nanotubes, they were designed based on the controlling kinetic diffusion procedure and Ostwald ripening process. Benefiting from boosted heterogeneous interfaces between TiN nanotubes and polydimethylsiloxane (PDMS), enhanced polarization loss relaxations were created, which could not only improve the depletion efficiency of EMWA, but also contribute to the optimized impedance matching at elevated temperature. Therefore, the TiN nanotubes/PDMS composite showed excellent EMWA performances at varied temperature (298–573 K), while achieved an effective absorption bandwidth (EAB) value of 3.23 GHz and a minimum reflection loss (RL_min_) value of − 44.15 dB at 423 K. This study not only clarifies the relationship between dielectric loss capacity (conduction loss and polarization loss) and temperature, but also breaks new ground for EM absorbers in wide temperature spectrum based on interface engineering.

**Supplementary Information:**

The online version contains supplementary material available at 10.1007/s40820-024-01381-w.

## Highlights

- The boosted heterogeneous interfaces in titanium nitride (TiN) nanotube/polydimethylsiloxane (PDMS) composite contributed to strong polarization loss relaxation ability.
- The TiN nanotubes/PDMS composite possessed both good impedance matching behavior and strong dielectric loss ability in wide temperature spectrum.
- The TiN nanotubes/PDMS composite exhibited excellent EMWA performances (effective absorption bandwidth value of 3.23 GHz and minimum reflection loss value of − 44.15 dB) at the varied temperature from 298 to 573 K.

**Supplementary Information:**

The online version contains supplementary material available at 10.1007/s40820-024-01381-w.

## Graphical abstract

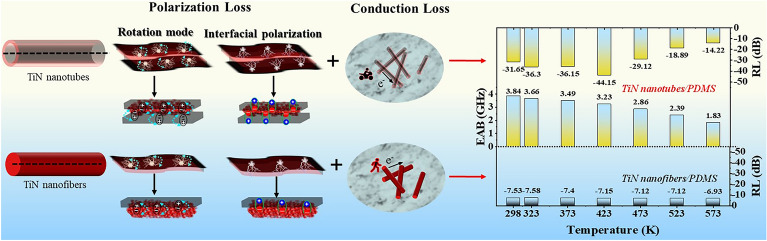

## Introduction

With the explosive development of electronic devices and wireless communication, the complex environment requires electromagnetic wave absorption (EMWA) materials that can cope with temperature changes [1–5]. The tremendous efforts have been devoted to exploring high-performance absorbers from two aspects: optimized impedance matching and strong EMW attenuation ability [6, 7]. According to the Debye theory, dielectric loss is function of temperature and consisted of polarization loss ($\varepsilon^{\prime\prime}_{p}$) and conduction loss ($\varepsilon^{\prime\prime}_{c}$):$\varepsilon^{\prime\prime} = \varepsilon^{\prime\prime}_{p} + \varepsilon^{\prime\prime}_{c} = (\varepsilon_{s} - \varepsilon_{\infty } )\frac{\omega \tau }{{1 + \omega^{2} \tau^{2} (T)^{2} }} + \frac{\sigma (T)}{{\varepsilon_{0} \omega }}$, in which $\varepsilon_{0}$, $\varepsilon_{\infty }$, $\varepsilon_{s}$, $\tau$, $\omega$, and $\sigma (T) = Ae^{ - E/2kT}$ is dielectric constant in vacuum, relative dielectric permittivity at the high frequency limit, static permittivity, relaxation time, angular frequency and temperature-dependent conductivity, respectively [7–9]. The electrons can hop and migrate in a conductive network under an EM field. With the rise of temperature, more electrons will be thermally activated to hop across the potential barrier and migrate faster, resulting in the boosted conductivity ($\sigma (T)$) and conduction loss ($\varepsilon^{\prime\prime}_{c}$) [7]. However, the enhanced conductivity will induce strong eddy current and lead to the impedance mismatching [7]. Differently, the interfacial polarization loss decreases with increasing the temperature, resulting in the weak loss ability and inferior EMWA performances [10]. According to the abovementioned analysis, the $\varepsilon^{\prime\prime}_{c}$ and $\varepsilon^{\prime\prime}_{p}$ with temperature dependence bring about the opposite temperature effect of dielectric loss. Therefore, it is imperative to have both good impedance matching and strong loss ability to achieve the high-performance EMWA in a fluctuating temperature environment [7, 8].

Recently, the dielectric composites with excellent temperature resistance characteristic and multiple loss mechanisms have garnered considerable attention [11]. For example, Cao et al. fabricated graphene/silica dioxide (SiO_2_) composites with a low filler content, which exhibited the stable impedance matching from 323 to 473 K due to the relative low conduction loss ratio [12]. Yin et al. prepared silicon carbide fibers/silicon nitride (SiC_f_/Si_3_N_4_) composite, which showed the relatively reliable high-temperature EMWA performances up to 873 K ascribed to the good impedance matching caused by improving the compensating effect of the decreased interfacial polarization loss [13]. Shi et al. synthesized titanium nitride/boron nitride (TiN/BN) nanocomposites that exhibited the steady broadband EMWA (maximum effective absorption bandwidth: EAB = 3.26 GHz, minimum EAB = 2.71 GHz) in the temperature range of 293–873 K, in which insulating BN both prevented TiN nanoparticles from agglomerating and provided additional interfacial polarization loss ability [14]. Besides components regulation, reasonable structural design was another effective strategy to improve impedance matching and achieve high-performance absorbers [15–27]. For example, Yin et al. constructed red blood cell like-mesoporous carbon hollow microspheres and sandwich-like reduced graphene oxide (RGO) and Si_3_N_4_ ceramic (RGO/Si_3_N_4_) composites, finding that the specific structure could result in boosting the interfacial polarization, which decreased with elevated temperature and compensated the increased $\varepsilon^{\prime\prime}_{c}$, dramatically contributing to the improvement of impedance matching at rising temperature [25, 26]. The optimized EMWA properties originated from the compensation effect of the decrease in polarization loss and increase in conduction loss at elevated temperature. Very recently, Jiang et al. created the pomegranate-like antimony-doped tin dioxide (ATO)/SiO_2_ spheres via a simple spray drying process, RL could reach − 47.3 dB at 573 K and EAB was 2.4 GHz, which was attributed to the effective local conductive network and abundant heterogeneous interfaces [3]. Evidently, abundant heterogeneous interfaces not only caused more intense polarization loss, but also modulated the EM parameters to improve the impedance matching, which provided an effective strategy for attenuating EMWs with temperature changes. Though developing a high-performance absorber with high polarization loss performance have been attracted, the inherent relationship between dielectric loss capacity (conduction loss and polarization loss) and temperature is still unclear. Up to now, there are few studies on exquisitely designed dielectric property, especially, the detailed variation of polarization loss mechanism in wide temperature spectrum [28].

Encouraged by the above consideration, the synergistic effect of components and structures contributes to the excellent EMWA performance at elevated temperature. Compared with other high-temperature ceramic material, such as silicon carbide (SiC), which needs doping approach to improve the loss ability of EMWs, TiN exhibits great potential as a high-temperature EMW absorber, attributed to the advantages of high melting point, high electrical and thermal conductivities, excellent environmental stability and EM coupling effect [29, 30]. Besides, the nanotubes architecture is deemed as the crucial branch of structure manipulation in increasing the EMWA due to its ultralow density, large interspace and ample interfaces, which can boost interfacial polarization, multiple scattering, and further increase the loss ability [15, 23]. In this work, to obtain satisfied impedance matching and strong EMWs attenuation capacity at high temperature, we ingeniously fabricated TiN nanotubes by electrospinning and thermal treatment method, according to the kinetic diffusion procedure and Ostwald ripening [31]. Compared to the TiN nanofibers/polydimethylsiloxane (PDMS) composite, the TiN nanotubes/PDMS composite exhibited the more abundant heterogeneous interfaces between TiN nanotubes and PDMS matrix inside the TiN nanotubes, which contributed to not only enhancing the interfacial polarization intensity, but also optimizing the impedance matching at elevated temperature (298–573 K). As a result, the responding TiN nanotubes/PDMS composite showed high-efficiency EMWA performances at the varied temperature (298–573 K), while achieved an EAB value of 3.23 GHz and a RL_min_ value of − 44.15 dB at 423 K, which indicated that constructing TiN nanotubes was an effective engineering to prepare high-performance EMW absorbers. Here, the interface engineering induced by well-designed nanotubes-structure enables as-prepared composites to achieve the strong dielectric losses, as well as the good impedance matching performance, which provides a new strategy for future high-temperature absorber design and refresh realization of EM loss mechanisms.

## Experimental Section

### Materials

Tetrabutyl titanate (Ti(OC_4_H_9_)_4_, TBT) was obtained from Tianjin Kemiou Chemical Reagent Co., Ltd; polyvinylpyrrolidone (PVP, K88-96) and iron acetylacetonate (Fe(C_5_H_7_O_2_)_3_) were supplied from Aladdin Reagent (Shanghai) Co., Ltd; polydimethylsiloxane (PDMS, SYLGARD(R)184) was purchased from Dow Corning Co., Ltd, respectively. All the raw materials were directly used without further purification.

### Preparation of TiN Nanotubes

TiN nanotubes were fabricated via a simple electrospinning and subsequent calcination process. 0.6 g PVP and 0.606 mmol Fe(C_5_H_7_O_2_)_3_ was added into a mixed solution containing 10.15 mL ethanol, 3.8 mL acetic acid and 3.8 mL TBT, followed by magnetic stirring for 30 min to assure the completely dissolution of PVP and form a light yellow spinning solution. It was sucked by a 5 mL medical syringe with a specific needle (outer diameter: 0.8 mm, inner diameter: 0.5 mm). Through the electrospinning apparatus with a 14.5 and − 2.5 kV voltage and a 0.25 mm min^−1^ solution feed rate, 20 cm receiving distance, the precursor was prepared, then dried at 150 °C for 24 h to obtain the spinning sample. Finally, the spinning sample was pretreated at 500 °C for 2 h with 1 °C min^−1^ heating rate under air atmosphere and was further nitrided at 900 °C for 4 h with 5 °C min^−1^ heating rate under NH_3_ atmosphere to produce TiN nanotubes. For comparison, TiN nanofibers with different hollow structure were also prepared by the similar route with different TBT amount (5.05 mL, 3.8 mL) and heating rate under air atmosphere (2, 0.5 °C min^−1^).

### Fabrication of TiN/PDMS Composites

The TiN/PDMS composites filled with 25 wt% TiN were fabricated by uniformly mixing TiN in PDMS matrix and corresponding curing agent under stirring at a speed of 2000 r min^−1^ for 10 min by centrifugal defoaming machine to ensure uniform dispersion, pouring into the mold (22.86 mm × 10.16 mm × 2 mm) and degassing at room temperature for 10 min in the vacuum oven to completely eliminate the gas, then placing in the oven for curing thoroughly at 80 °C for 3 h.

### Characterization

The morphology of TiN with different hollow structure was observed by scanning electron microscopy (SEM, Carl Zeiss Gemini 500) and transmission electron microscopy (TEM, Hitachi H-8100). Their structure and composition information were analyzed by the X-ray diffractometer (XRD, Bruker D8-Advance) and Raman spectra (Horiba LabRAM, laser excitation wavelength: 532 nm; exposure time: 3 s). The EM parameters of the corresponding TiN/PDMS composites were obtained by a vector network analyzer (VNA) (Ceyear, 3672B-S) using the wave-guide method at the varied temperatures (298–573 K) in the X-band, as shown in Fig. S1. The TiN/PDMS composites were positioned vertically in the center of test chamber in Ar atmosphere with a heating rate of 5 °C min^−1^, and each test temperature was held for 3 min to acquire accurate EM parameters, then the next set temperature point was followed. Moreover, as a comparison, both the EM parameters and optical photographs of PDMS before and after testing were also provided (as exhibited in Fig. S2), and they are almost consistent ($\varepsilon^{\prime}$ and $\varepsilon^{\prime\prime}$ values were ≤ 3 and 0.1, respectively), proving that the prepared material possess good thermal stability.

## Results and Discussion

### Structural and Morphological Properties

**Fig. 1:**
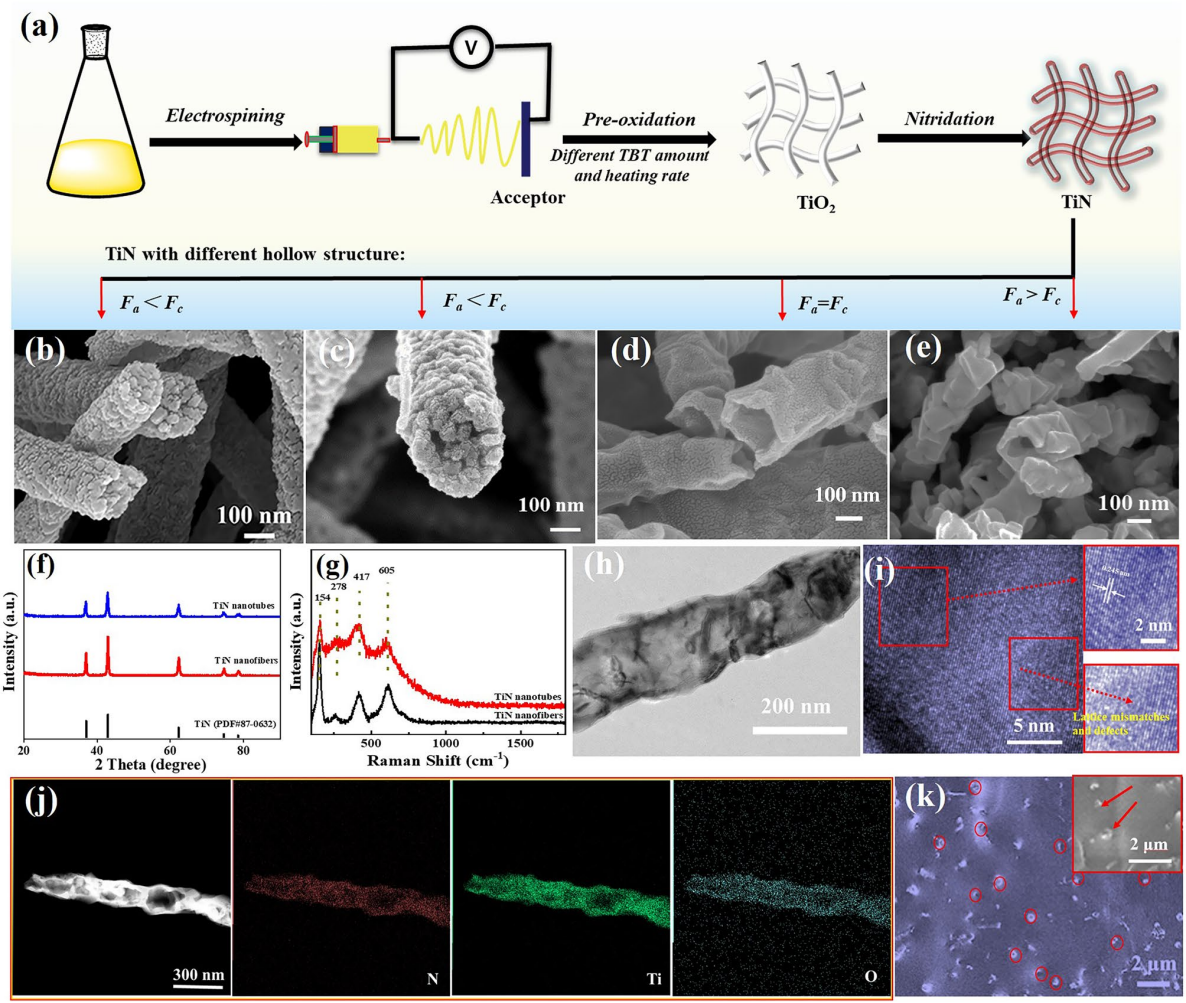
Schematic illustration of the fabrication process of **a** TiN and **b–e** SEM images of TiN with different hollow structure, which contained different TBT amount and heating rate of pre-oxidation under air atmosphere: **b** 5.05 mL, 2 °C min^−1^, **c** 3.8 mL, 2 °C min^−1^, **d** 3.8 mL, 1 °C min^−1^, **e** 3.8 mL, 0.5 °C min^−1^. XRD pattern (**f**) and Raman spectra (**g**) of TiN nanotube and TiN nanofiber, TEM images (**h**,** i**) and elemental mapping (**j**) of TiN nanotubes and cross-sectional SEM images (**k**) of TiN nanotubes/PDMS composites

To obtain the controllable manipulation of interface engineering, TiN nanotubes are designed, as shown in Fig. 1a. Beginning with the thought of creating and triggering more heterogeneous interfaces, TiN nanotubes are dexterously devised via changing the TBT amount and heating rate of pre-oxidation temperature. The TiN nanotubes are ultimately harvested by facile electrospinning and calcination methods with an aim of exchange reaction. Firstly, the uniform precursor nanofibers are prepared by an electrospinning method. Secondly, TiN nanotubes are created by the pre-oxidation and nitriding process, which are closely related with the decomposition of PVP, formation of TiN layer and diffusion of metal cations. Using 5.05 mL TBT and 2 °C min^−1^ heating rate of pre-oxidation, the produced nanofibers are comprised of TiN nanoparticles, as shown in Fig. 1b. With the decrease in TBT (3.8 mL) and invariability of heating rate of pre-oxidation (2 °C min^−1^), the corresponding TiN nanofibers emerge a partial void (Fig. 1c). When the heating rate is 1 °C min^−1^, the product becomes nanotube structure with the ~ 25 nm thin wall (Fig. 1d). Continue to reduce the heating rate (0.5 °C min^−1^), TiN nanofibers become partial void (Fig. 1e). The result reveals that the formation of nanotubes is predominantly influenced by the TBT amount and heating rate of pre-oxidation, which is closely related with Ostwald ripening procedure and kinetic diffusion, leading to the in situ formation of TiN layer and diffusion of metal cations [31]. Reasonably, two opposing forces are acting simultaneously on the wall: contraction force (*F*_c_) and adhesive force (*F*_a_), which derives from the thermal degradation of organic species to facilitate the shrinkage of wall, and the rigid surface that restrains the inward shrinkage, respectively [31, 32]. Spontaneously, through applying the suitable TBT amount and heating rate of pre-oxidation, scilicet *F*_a_ = *F*_c_, the wall will be created due to the interaction and dynamic equilibrium between *F*_c_ and *F*_a_ [31]. However, if TBT amount and heating rate of pre-oxidation is so much and fast, more TiN nanocrystals continuously diffuse outward and prefer to aggregate into larger particles, as shown in Fig. 1b, c, the *F*_c_ increases and the dynamic equilibrium is broken, thus the 1D nanofiber or/with partial void is generated [33]. Contrariwise, when the heating rate of pre-oxidation is slow, the decomposition of PVP is low, leading to the less *F*_c_, which results in the nanofiber with partial void and small crystalline grain (Fig. 1e). Therefore, by rational controlling the Ostwald ripening and kinetic diffusion procedure, both the growth of TiN nanocrystalline and the diffusion kinetics of Ti element could be regulated, which contribute to the formation of nanotube structure [31].

The crystalline structures and components of as-synthesized TiN are revealed, based on XRD patterns and Raman spectra. The diffraction peaks at 36.8°, 42.8°, 62.1°, 74.4°, and 78.3° are indexed to the (111), (200), (220), (311), and (222) planes of face-centered cube TiN phase (PDF#38-1420) (Fig. 1f), and no diffraction peaks are detected, implying the formation of pure TiN nanocrystalline. As to the Raman spectra (Fig. 1g), the distinctive peaks located at 149, 311, 457, and 675 cm^−1^ are assigned to the nonstoichiometric TiN, no other peaks are identified, which agrees with the XRD result [29]. Figure 1h, j shows the TEM images of the representative TiN nanotubes, and the nanoparticles are uniformly distributed in the surface to form the wall, which contains the homogeneous distribution of Ti, N, and O elements. Figure 1i exhibits the lattice fringes of 0.207 nm, which can be indexed to (111) plane of TiN, and the obvious lattice mismatches and defects, such as lattice deformation, lattice dislocation and discontinuous fringe, are found [34]. The escape of gases during the carbonization process tends to cause a substantial number of defects and lattice mismatches, prompting the generation of polar units due to the accumulation of charges at the interfaces induced by the diverse electrical conductivities [35]. Thereby, ample heterogeneous interfaces are customized in TiN nanotubes, which are functioned as “polarization centers”, triggering strong polarization loss, which is conducive to the enhancement of polarization loss [36, 37]. Besides, a cross-sectional SEM image (Fig. 1k) of TiN nanotubes/PDMS composites is described, which confirms the discrete distribution of TiN nanotubes in the PDMS matrix. Moreover, the PDMS matrix in the nanotubes could further increase the heterogeneous interfaces between TiN nanotubes and PDMS matrix. Thus, the interface engineering is not only beneficial to enlarge the heterogeneous interfaces between filler and matrix and improve the interfacial polarization loss, but also conducive to enhance the impedance matching, leading to the more EMWs to interact with the absorber and further be attenuated, which contributes to obtaining the optimal EMWA performances.

### Microwave Absorption Properties

**Fig. 2:**
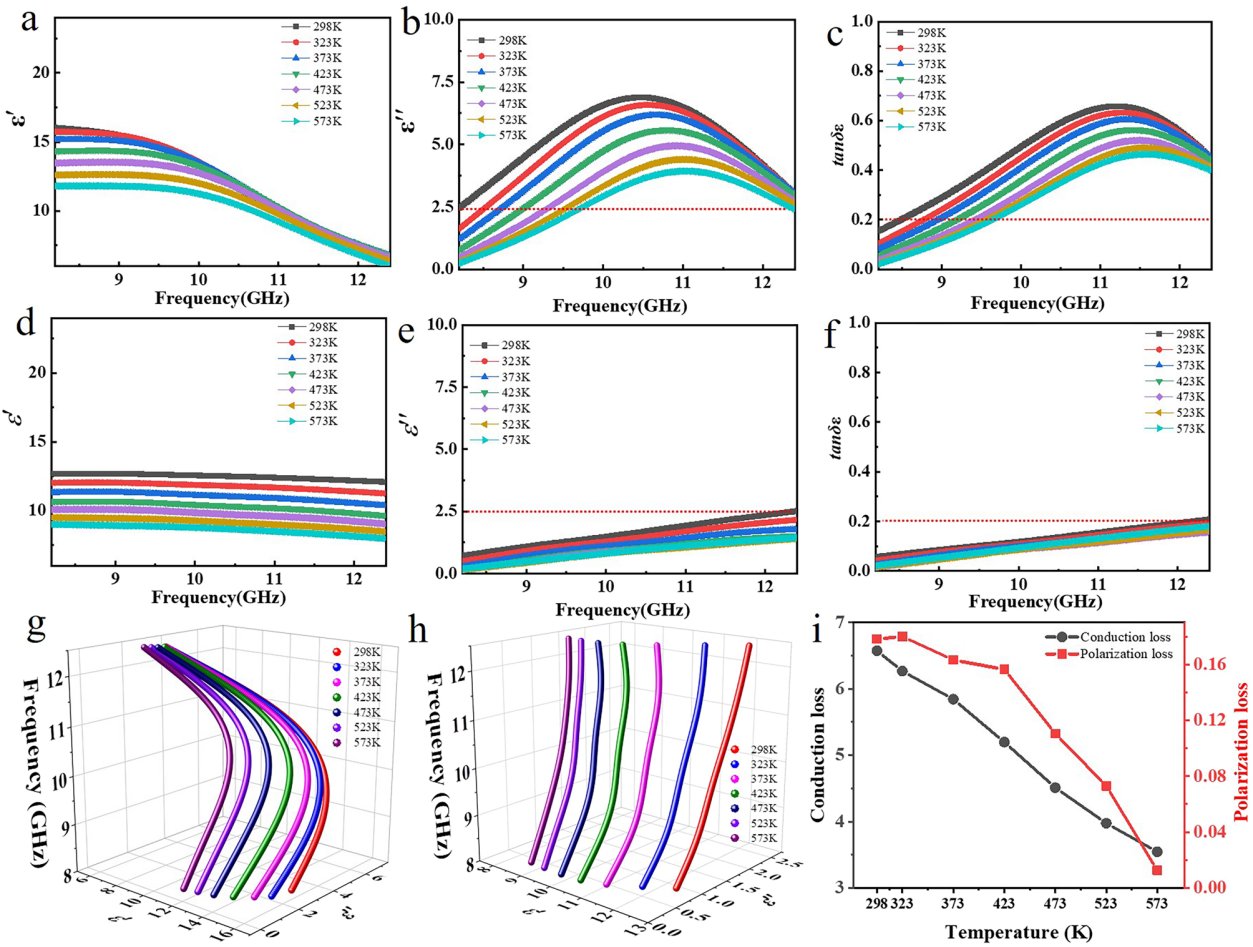
**a**,** d** *ε′*, **b**,** e** *ε*″, **c**,** f** tan*δε* and **g**,** h** Cole–Cole curves of TiN nanotubes/PDMS (**a**–**c**, **g**) and TiN nanofibers/PDMS (**d–f, h**) versus frequency at 298–573 K. **i** The fitted average $\varepsilon^{\prime\prime}_{c}$ and $\varepsilon^{\prime\prime}_{p}$ of TiN nanotubes/PDMS versus 10–11 GHz at different temperature

Considering that the temperature and frequency response behavior of polarization relaxation process, to confirm the relationship between polarization loss and EMWA performances, the influence of interface engineering on EM parameters (*ε′* and *ε*″) of TiN/PDMS composites are investigated, while *ε′* and *ε*″ imply the polarization and dielectric loss, respectively [38]. As shown in Fig. 2, *ε′* values of TiN nanotubes/PDMS composite present the increased phenomenon and noticeable frequency dissipation behavior (Fig. 2a), compared with those of TiN nanofibers/PDMS composite (Fig. 2d), revealing the boosted polarization [35, 38]. Specially, the polarized platform of TiN nanotubes/PDMS composite (Fig. 2a) in about 8.2–9.5 GHz induced by the abundant heterogeneous interfaces implies the strong polarization [38]. It is mainly due to the decrease in polarization hysteresis, indicating the typical polarization process [39]. Meanwhile, the frequency-dependence of *ε″* values also displays obvious difference. One broad dielectric relaxation peak appears in TiN nanotubes/PDMS composite, while no evident relaxation peaks could be found in TiN nanofibers/PDMS system, proving the link between composition/microstructure and strong polarization loss. It is mainly attributed that the TiN nanotubes can produce more heterogeneous interfaces between filler (TiN) and matrix (PDMS) inside the nanotube, which is beneficial to the generation of polarization loss [17]. And, dielectric property difference between TiN and PDMS also lures the interfacial polarization [28]. The frequency-dependence of *ε″* values of TiN nanotubes/PDMS composite exhibits an evident lag phenomenon, meaning the boosted lagging of polarization and the corresponding strong dielectric loss, which is attributed that the polar unit dissipates more EM energy to overcome the rotational resistance and intrinsic energy [19]. With the incremental temperature, the improved thermal motion enhances the orientation rotation of polar unit, therefore leading to the reduced polarization loss [19]. Meanwhile, the decrease in the energy required for charge movement at an elevated temperature makes polar unit movement more powerful and easier to keep up with changes in the EM field. Thus, according to the above analysis, both *ε″* values and dielectric loss character decrease with the increased temperature.

To verify the relaxation behaviors further, Cole–Cole curves ($\varepsilon^{\prime} - \frac{{\varepsilon_{s} + \varepsilon_{\infty } }}{2})^{2} + (\varepsilon^{\prime\prime})^{2} = (\frac{{\varepsilon_{s} - \varepsilon_{\infty } }}{2})^{2}$) are also provided, as shown in Fig. 2g, h, in which each semicircle is associated with one Debye relaxation process [40, 41]. Compared with those of TiN nanofibers/PDMS composite, the Cole–Cole curves of TiN nanotubes/PDMS composite maintain the perfectly smooth semicircle shape in 298–573 K without tail attributed to the increased effective heterogeneous interfaces and enhanced interfacial polarization intensity, indicating the strong polarization relaxation loss character [42]. Besides, the ${\text{tan}}\delta \varepsilon$ of TiN nanotubes/PDMS (0.02–0.66) is larger with respect to that of TiN nanofibers/PDMS (0.02–0.21) in the whole measured frequency and temperature range, indicating the improved dielectric loss ability derived from the interface engineering, as shown in Fig. 2c, f [37].

To illustrate the inherent contribution of polarization loss ($\varepsilon^{\prime\prime}_{p}$) and conduction loss ($\varepsilon^{\prime\prime}_{c}$) on the dielectric loss ($\varepsilon^{\prime\prime}$), Fig. 2i compares the average $\varepsilon^{\prime\prime}_{c}$ and $\varepsilon^{\prime\prime}_{p}$ values of TiN nanotubes/PDMS composite located at 10–11 GHz, which corresponds the relaxation peaks. The $\varepsilon^{\prime\prime}_{p}$ and $\varepsilon^{\prime\prime}_{c}$ has been determined by the nonlinear least squares fitting method. The model function can be described as follows [43]:

1 $$ \varepsilon = \varepsilon_{\infty } + (\varepsilon_{s} - \varepsilon_{\infty } )/(1 + \omega^{2} \tau^{2} ) - i((\varepsilon_{s} - \varepsilon_{\infty } )\omega \tau /(1 + \omega^{2} \tau^{2} ) + \sigma /(\omega \varepsilon_{0} )) $$

To fit $\varepsilon^{\prime\prime}_{p}$ and $\varepsilon^{\prime\prime}_{c}$ as accurate as possible, the data are divided into 20 groups, and the $\varepsilon_{s}$, $\varepsilon_{\infty }$, $\tau$ and $\sigma$ are fitted firstly, corresponding to the method adopted by some research [19, 26, 43]. It is found that the average $\varepsilon^{\prime\prime}_{c}$ and $\varepsilon^{\prime\prime}_{p}$ values decline with the elevated temperature from 298 to 573 K, according with abovementioned analysis of $\varepsilon^{\prime\prime}$ values. Meanwhile, it is obvious that $\varepsilon^{\prime\prime}_{p}$ values gradually decrease at 298–423 K and dramatically reduce exceeded at 473 K. When the temperature is 298 K, orientation rotation of polar unit requires enough high energy to overcome rotational resistance and intrinsic energy, leading to the severe polarization lag and producing strong polarization loss in TiN nanotubes/PDMS composite, thereby the $\varepsilon^{\prime\prime}_{p}$ value is high. As the temperature elevates (≥ 473 K), the external environment endows polar unit more energy, which makes it easier to overcome the orientation resistance and intrinsic energy. Therefore, the polarization lagging phenomenon could be alleviated, which results in a decreased contribution ratio on dielectric loss.

**Fig. 3:**
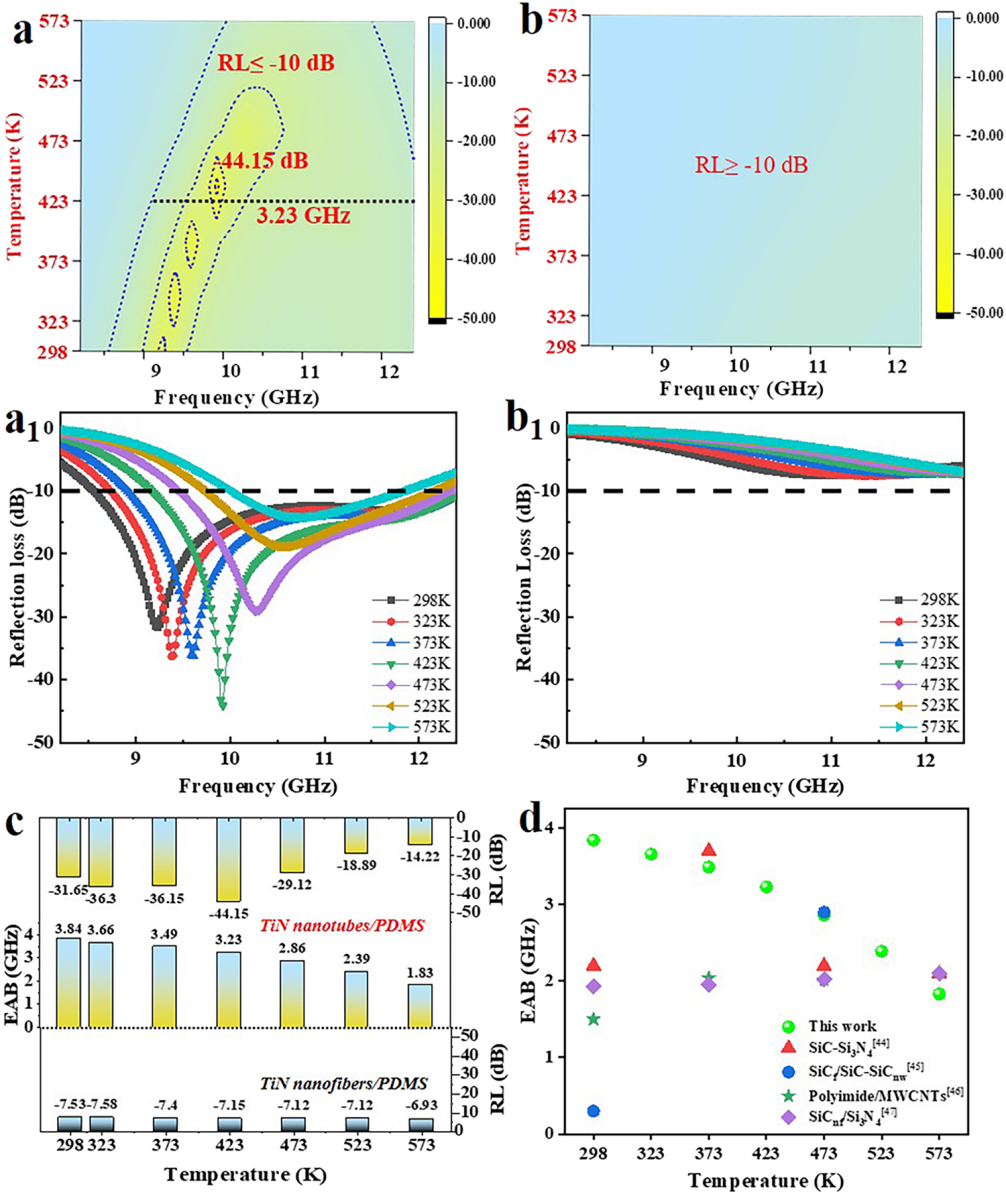
Microwave absorption properties (**a**–**b**_**1**_) of TiN nanotubes/PDMS (**a, a**_**1**_) and TiN nanofibers/PDMS (**b, b**_**1**_), EAB and RL_min_ values (**c**), and comparison of EMWA performances with reported efficient absorbers (**d**) [44–47]

The EMWA performances are calculated based on the transmission line theory [48–50]:

2 $$ Z_{in} = Z_{o} (\mu_{r} /\varepsilon_{r} )^{1/2} \tanh \left[ {j(2\pi fd/c)(\mu_{r} \varepsilon_{r} )^{1/2} )} \right] $$

3 $$ {\text{RL}}({\text{dB}}) = 20\log \left| {(Z_{in} - Z_{o} )/(Z_{in} + Z_{o} )} \right| $$

where *Z*_*in*_ and *Z*_*0*_ represent the input impedance of the absorber and impedance of free space, *f*, *d* and *c* correspond to microwave frequency, thickness of the absorber and velocity of light, respectively; and $\varepsilon_{r}$ ($\varepsilon_{r} = \varepsilon^{\prime} - j\varepsilon^{\prime\prime}$) and $\mu_{r}$ ($\mu_{r} = \mu^{\prime} - j\mu^{\prime\prime}$) refer to the relative complex permittivity and permeability, respectively. When further investigating the impact of interface engineering on the performances of EMWA versus frequency and temperature, it could be found that TiN nanotubes/PDMS composite (Fig. 3a, a_1_) exhibits relatively excellent EMWA capacity at 298 K, and the RL_min_ value reaches − 31.64 dB at 9.23 GHz with 2.1 mm, and the EAB is 3.84 GHz (8.56–12.4 GHz). Moreover, the TiN nanotubes/PDMS composite possesses a high absorption belt with one excellent EMWA ‘islands’ at 423 K, while the EAB can reach about 3.23 GHz and the optimal RL can be up to − 44.15 dB, demonstrating the gratifying EMWA performance within a broad temperature range (Fig. 3b). Yet, the RL_min_ value of TiN nanofibers/PDMS composite is only − 7.53 dB at 298 K (Fig. 3c) due to the inferior impedance matching and weak loss ability. Compared with those of reported efficient absorbers at the varied temperature, which are summarized in Fig. 3d, the TiN nanotubes/PDMS composite appears more significant advantages both in the EAB and wide temperature spectrum.

**Fig. 4:**
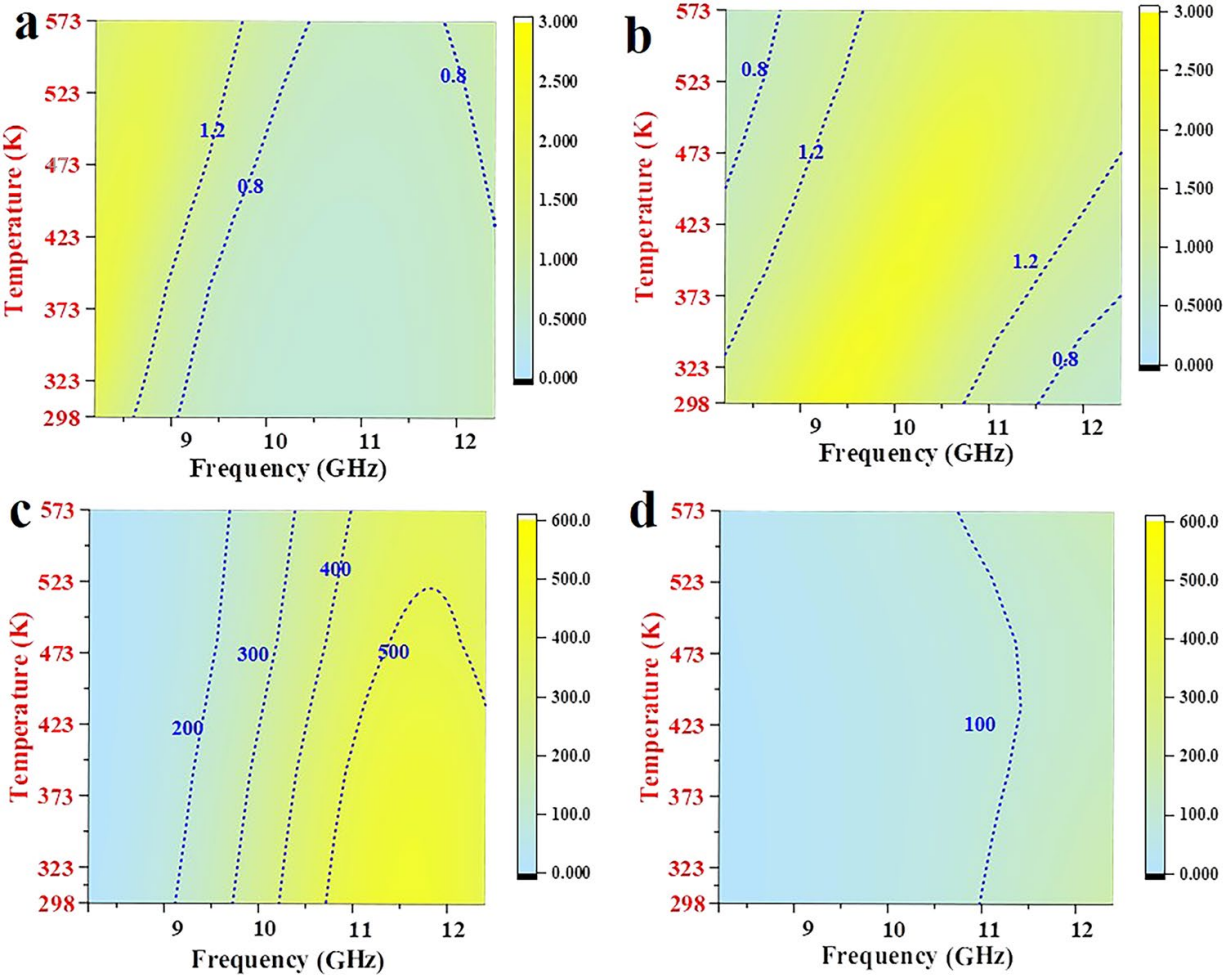
**a, b** Impedance matching and **c, d** attenuation constant of TiN nanotubes/PDMS (**a, c**) and TiN nanofibers/PDMS (**b, d**) composites

The impedance matching ($Z = \left| {Z_{in} /Z_{o} } \right|$) and attenuation ability $\sqrt {(\mu^{\prime\prime}\varepsilon^{\prime\prime} - \mu^{\prime}\varepsilon^{\prime}) + \sqrt {(\mu^{\prime\prime}\varepsilon^{\prime\prime} - \mu^{\prime}\varepsilon^{\prime})^{2} + (\mu^{\prime}\varepsilon^{\prime\prime} + \mu^{\prime\prime}\varepsilon^{\prime})^{2} } }$) of absorbers are two determinants to regulate EMWA performances [51–56]. When the *Z* gets closer to 1, it means that the more EMWs can enter the interior of the material, revealing the generation of stronger reflection loss. Meanwhile, when the $\alpha$ is higher, it indicates the stronger EMWs attenuation ability. A good balance between $Z$ and $\alpha$ contributes to producing EMWA performances. Compared with those of TiN nanofibers/PDMS composite (Fig. 4b, d), Fig. 4a, c exhibits the good impedance matching and large attenuation constant of TiN nanotubes/PDMS composite. The nanotube structure promotes better penetration of EMWs into the material, thereby serving as an “effective medium” for impedance matching [15]. Besides, the electrical characteristic also varies remarkably at the grain boundaries of heterogeneous interfaces, the diverse charge density functions as the condition to create capacitor-like structure and forms interfacial polarization with the synergism of heterogeneous interfaces [15]. Apparently, the strategy of interface engineering induces the ample heterogeneous interfaces, which can cause the multiple scattering routes of EMWs and serve as traps to capture and consume EMWs, as well as enlarge the electrical characteristic, ameliorating the polarization relaxation and conduction losses, effectively enhancing the absorptive capacity and EMWA performances [15, 57].

**Fig. 5:**
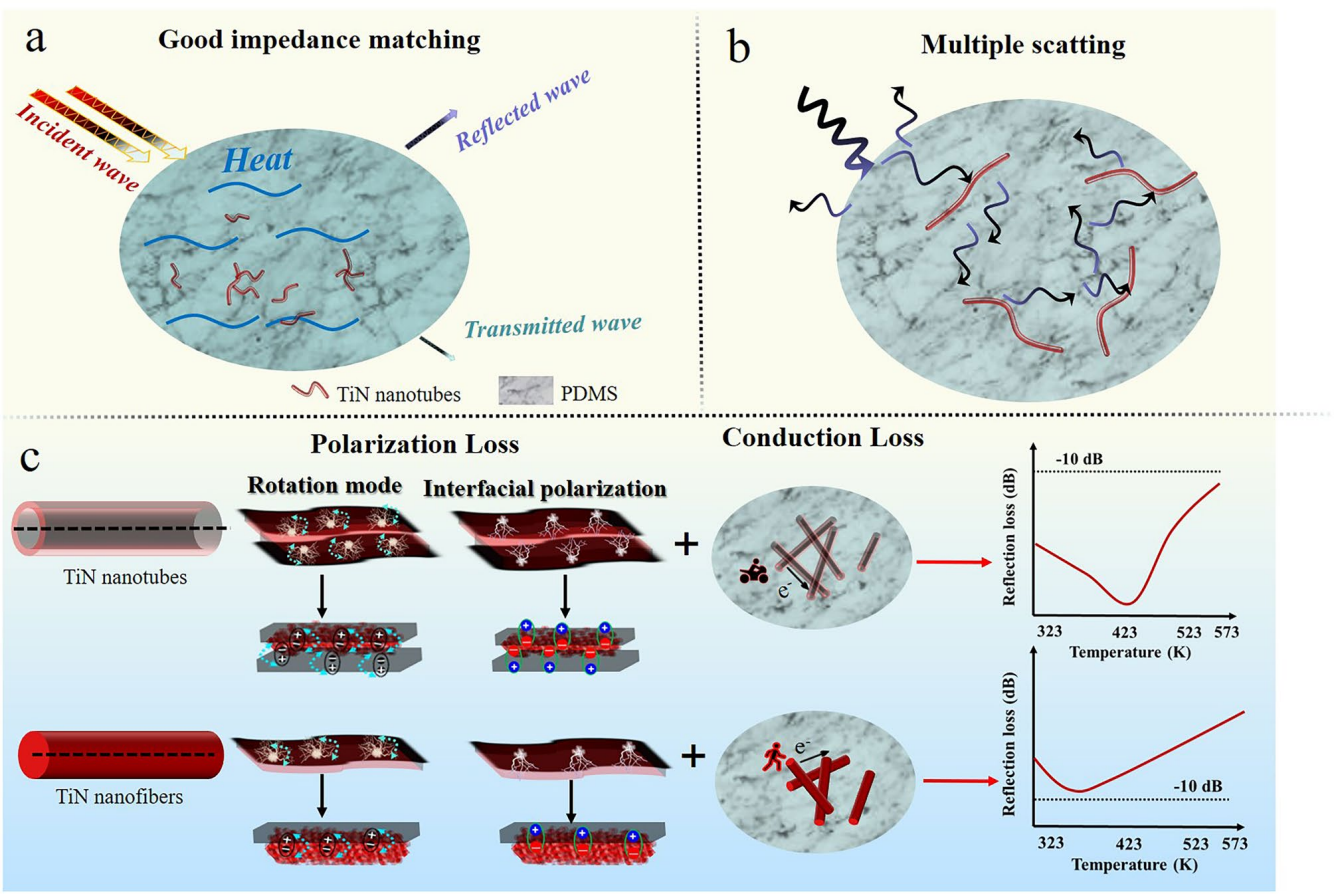
Mechanism of TiN nanotubes/PDMS for EMWs attenuation: **a** impedance matching, **b** multiple scattering, **c** polarization loss and conduction loss

The interface engineering modulates the polarization loss in the TiN nanotubes/PDMS composite, which endows the optimized impedance matching and enhanced attenuation ability. Specifically, TiN nanofibers/PDMS composite with poor dielectric parameters and EMWA performances shows a weakened response to EMWs. When nanotube exists in the TiN nanotubes/PDMS composite, the abundant heterogeneous interfaces are created. The boosted polarization loss induced by interface engineering confers the enhanced loss ability and strong EM response. Excellent EMWA performances are mainly due to the following factors: (1) the better impedance matching and stronger attenuation ability of TiN nanotubes/PDMS composite allows more EMWs to enter the interior of TiN nanotubes/PDMS composite and be further attenuated (Fig. 5a), which is conductive to enhance the EMWA performances [58]. (2) The incident EMWs are trapped in the TiN nanotubes/PDMS composite and can be further consumed by the multiple scatting effect until they are exhausted (Fig. 5b), contributing to improving the loss ability [20, 59]. Meanwhile, the construction of TiN nanotubes is beneficial to increase the contact area between EMWs and absorber, and further boost the loss ability. (3) Compared with TiN nanofibers, we propose TiN nanotube microstructure to produce more heterogeneous interfaces, which can generate charge redistribution, transfer, and accumulation, hence contributing to reinforcing the conduction loss and interfacial polarization [60, 61] (Fig. 5c). The rational design of TiN nanotube, in which PDMS matrix can enter into the nanotube to provide the abundant heterogeneous interfaces between conductive TiN and insulating PDMS matrix, producing the enhanced interfacial polarization, which is beneficial to increase the loss capacity [26, 62–66]. Meanwhile, the polar unit requires more EM energy to overcome the rotational resistance and intrinsic energy, prompting the improvement of polarization loss and loss ability. Undoubtedly, interface engineering strategy elaborates the relationship between loss ability and variable temperature, and grants TiN nanotubes/PDMS composite more loss mechanism to obtain the high-performance EMWA.

## Conclusions

In summary, we have presented the fabrication of TiN nanotubes to investigate the underlying correlation between polarization loss and variable temperatures, wherein temperature-dependent polar units enable modulation of polarizability. By the design of interface engineering, the strong polarization loss was obtained, which was a slight variation at 298–423 K, yet decreased dramatically exceeded 423 K. As a result, excellent EMWA performances with optimal RL of -44.15 dB and broad EAB of 3.23 GHz at 423 K were achieved ascribed to both good impedance matching and strong loss ability. Here, the nanotube with ample heterogeneous interfaces serve as a pivotal structure in the improvement of interfacial polarization, which provides a strategy for polarization control of EMWA performance and makes it possible to further investigate the effect of the dielectric polarization behavior.

## Supplementary Information

Below is the link to the electronic supplementary material.

Supplementary file1 (DOCX 1723 KB)
