# Supplementary material for: Interface Engineering of Titanium Nitride Nanotube Composites for Excellent Microwave Absorption at Elevated Temperature
Source: Nanomicro Lett. 2024 Apr 4;16:168. doi: 10.1007/s40820-024-01381-w (PMC10994892; doi:10.1007/s40820-024-01381-w)
Supplement: Supplementary file 1 — Supplementary file1 (DOCX 1723 KB) [file 40820_2024_1381_MOESM1_ESM.docx]

Supporting Information for

**Interface Engineering of Titanium Nitride Nanotube Composites for Excellent Microwave Absorption at Elevated Temperature**

Cuiping Li^1, 2, #^, Dan Li^1, 2, #^, Shuai Zhang^2^, Long Ma^1, 2^, Lei Zhang^1,^ *, Jingwei Zhang^2^, Chunhong Gong^1, 2,^ *

^1^ College of Chemistry and Molecular Sciences, Henan University, Kaifeng 475004, China

^2^ National & Local Joint Engineering Research Center for Applied Technology of Hybrid Nanomaterials, Henan University, Kaifeng 475004, China

^#^ Cuiping Li and Dan Li contributed equally to this work.

*Corresponding author. E-mail: [gong@henu.edu.cn](mailto:gong@henu.edu.cn) (Chunhong Gong) and [zhangl@henu.edu.cn](mailto:zhangl@henu.edu.cn) (Lei Zhang)

**Supplementary Figures**


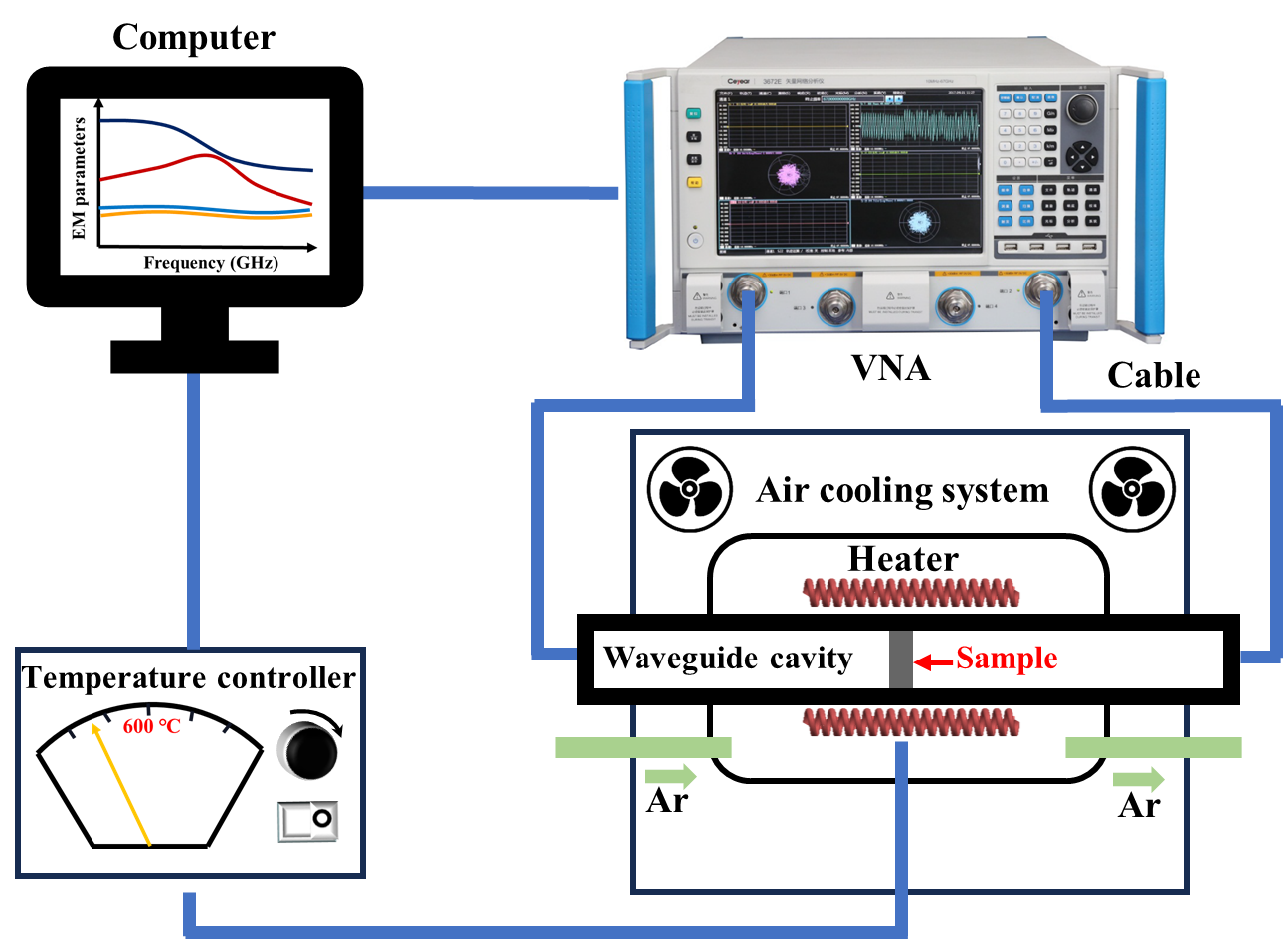


**Fig. S1** Measurement of the high-temperature EM properties

**Fig. S2** The EM parameters **a–d** and optical photographs **e** of PDMS matrix versus frequency at 298–573 K: (**a, b**) first test results, (**c, d**) second test results
